# Characterization of the human endogenous retrovirus K Gag protein: identification of protease cleavage sites

**DOI:** 10.1186/1742-4690-8-21

**Published:** 2011-03-23

**Authors:** Benjamin Kraus, Klaus Boller, Andreas Reuter, Barbara S Schnierle

**Affiliations:** 1 Paul-Ehrlich-Institut, Paul-Ehrlich-Strasse 51-59, 63225 Langen, Germany

## Abstract

**Background:**

Viral genomes of the human endogenous retrovirus K (HERV-K) family are integrated into the human chromosome and are transmitted vertically as Mendelian genes. Although viral particles are released by some transformed cells, they have never been shown to be infectious. In general, gammaretroviruses are produced as immature viral particles by accumulation of the Gag polyproteins at the plasma membrane, which subsequently bud from the cell surface. After release from the cell, Gag is further processed by proteolytic cleavage by the viral protease (PR), which results in morphologically mature particles with condensed cores. The HERV-K Gag polyprotein processing and function has not yet been precisely determined.

**Results:**

We generated a recombinant poxvirus, encoding the human endogenous retrovirus K consensus gag-pro-pol genes (MVA-HERV-K_con_) and obtained high levels of HERV-K Gag expression. The resulting retroviral particle assembled at the plasma membrane, as is typical for gammaretroviruses; and immature as well as mature retrovirus-like particles (VLPs) were observed around the infected cells. VLPs were purified, concentrated and separated by two-dimensional gel electrophoresis. The HERV-K Gag fragments were identified by mass spectroscopy and N-terminal sequencing which revealed that HERV-K Gag is processed into MA, a short spacer peptide, p15, CA and NC.

**Conclusion:**

The cleavage sites of HERV-K Gag were mapped and found to be highly conserved among HERV-K genomes. The consensus HERV-K gag gene used in this study is known to support viral, infectivity [1], and thus the cleavage sites that were mapped in this study for all the Gag components are relevant for HERV-K infectivity.

## Background

Human endogenous retroviruses (HERVs) are relics of evolutionary ancient viral infection events which involved insertion into the germ line and are now transmitted vertically. These retroviral genomes are chromosomally integrated in all nucleated cells of an individual and their sequences, including solitary LTRs, comprise about 8% of the human genome. HERVs are classified by the single letter amino acid code for the tRNA specific to the primer-binding site (PBS) used to initiate reverse transcription. At present, 11 distantly related HERV groups with a tRNA lysine (K) PBS exist (reviewed in [2]). One of these, the HERV-K/HML-2(hom) group is the only known endogenous retrovirus group encoding all structural and enzymatic proteins (proteins encoding the viral core [Gag], UTPase/protease [PR], polymerase [Pol], RNaseH, integrase [Int]), and envelope [Env]) and the accessory protein Rec with functional similarity to the HIV Rev protein [3]. In general, HERV-K gene expression is repressed in somatic cells; however, reactivation of HERV-K proviruses coding for all viral proteins has been described for human teratocarcinomas [4], as well as for melanomas [5], and ovarian cancer [6], [7]. Although full-length genomic mRNA and viral particles are detectable, HERV-K has never been shown to be infectious and the viral genome is only known to be transmitted via the germ line. However, the HERV-K family may have replicated less than 1 million years ago and a consensus HERV-K sequence has been constructed that likely resembles the progenitor of HERV-K that entered the human genome within the last few million years. Infectious particles could be generated from this consensus HERV-K provirus sequence [1], [8]. This raises concerns that recombination between different HERV-K genomes might be able to form infectious viral genomes able to reinfect human cells, especially germ cells.

Retroviral Gag polyproteins play a central role in viral particle formation and are responsible for particle assembly, release and infectivity. Gag itself is sufficient for the formation of virus-like particles (VLPs). It is first synthesized as a polyprotein and is further processed in viral particles by the viral protease (PR) to produce the major structural proteins MA (matrix), CA (capsid) and NC (nucleocapsid). This genetic organization as a polyprotein precursor that is subsequently cleaved exploits the protein-protein interactions needed for virion assembly to ensure the incorporation of the enzymes needed for replication, maturation and infection. Mutation of the retroviral PR prevents cleavage but does not prevent assembly of Gag at the plasma membrane and virus release; however, the resulting particles remain immature, with a changed morphology that never shows collapsed cores, and are not infectious.

Cell associated HERV-K particles budding from human teratocarcinoma cell lines have been shown to exist exclusively as morphologically immature particles [9]. However, a functional protease has been detected [10]. This raises the possibility that a defect in cleavage is caused by mutated cleavage sites. Therefore, we have characterized here the HERV-K Gag protein processing based on the consensus HERV-K genome sequence [1].

## Results

### HERV-K Gag processing is mediated by the viral protease

**Figure 1:**
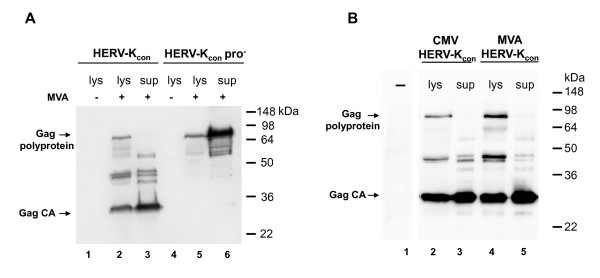
**HERV-K Gag polyprotein cleavage requires a functional retroviral protease**. Western blot analysis of HERV-K Gag, expressed by transient vaccinia virus-driven expression. HERV-K Gag was detected with the HERV-K CA-specific monoclonal antibody mix HERMA [9] and an HRP-coupled anti-mouse antibody followed by ECL detection (Amersham, Freiburg). A: Lys: cell lysates; sup: concentrated cell culture supernatants. Lanes 1-3: HERV-K Gag expression with a functional protease; Lanes 4-6: HERV-K Gag expression without protease; +: plasmid transfection with additional MVA infection. Positions of the Gag polyprotein and CA are indicated. B: Lys: cell lysates; sup: concentrated cell culture supernatants. Lane 1: lysate of untreated 293T cells; lanes 2-3: plasmid driven HERV-K Gag expression, samples prepared 48 hrs after transfection; Lanes 4-5: MVA-HERV-K_con _driven HERV-K Gag expression, samples prepared 24 hrs after infection. The loading volume was one-third of the volume used in lanes 1, 2 and 3.

Processing of retroviral Gag polyproteins in immature viral particles is mediated by the viral protease (PR) and induces a reorganization of the internal virion structure, termed maturation, which becomes morphologically visible as collapse of the viral core. Mutation or deletion of PR prevents cleavage but does not prevent assembly, resulting in immature particles. A functional protease has been described for HERV-K [10]. To analyze a potential defect in Gag cleavage and to obtain high amounts of HERV-K particles, HERV-K Gag expression with the help of the modified vaccinia virus Ankara (MVA) was employed. The consensus HERV-K Gag-PR-Pol gene [1] was cloned into the MVA expression vector pIII-mH5 [11] generating the plasmid pIII-HERV-K_con_, where expression is controlled by a strong early/late promoter. In addition, we constructed a protease-deficient variant of that construct, named pIII-HERV-K_con_pro^-^. The transfer plasmids pIII-HERV-K_con _and pIII-HERV-K_con_pro^- ^were transfected into HEK 293T cells, and these were infected with MVA to induce transient expression of the HERV-K gag/pol genes controlled by the vaccinia virus specific promoter. Subsequent Western blot analysis of cell lysates (lys) and concentrated supernatants (sup) revealed that HERV-K Gag/Pol was expressed after MVA infection (Figure 1A, lanes 2, 3, 5 and 6) and particles could be pelleted by ultracentrifugation from supernatants of these cells (lanes 3 and 6). Particles derived from pIII-HERV-K_con _contained mainly processed Gag, since the Gag precursor could only be detected in cell lysates and not in the supernatants (Figure 1A, lanes 2 and 3). However, only the Gag precursor could be detected in cell lysates and viral particles from pIII-HERV-K_con_pro^-^-transfected cells (Figure 1A, lanes 5 and 6). Consequently, processing of HERV-K Gag was dependent on the presence of the functional retroviral protease and MVA infection was not responsible for cleavage products.

### HERV-K particles are released from cells infected with recombinant HERV-K Gag/Pol-expressing MVA

After showing the value of this expression system for HERV-K Gag processing analysis, we generated a recombinant MVA stably expressing the HERV-K gag/pol gene (MVA-HERV-K_con_). The recombinant virus was constructed as described previously, using K1L selection [11]. Quality control by PCR and Western blot analysis confirmed the proper expression of HERV-K Gag/Pol in cells infected with MVA-HERV-K_con _(Figure 1B, lanes 4 and 5). To further demonstrate that MVA does not alter the cleavage pattern of Gag, we additionally analyzed cell lysates and concentrated viral particles from HEK 293T cells transfected with the HERV-K Gag-PR-Pol expression plasmid which expresses HERV-K Gag/Pol driven by a CMV promoter 48 hrs after transfection [1]. Fragments, representing Gag and capsid or partial cleavage products thereof had the same size independently if HERV-K Gag/pol was expressed in the presence or absence of MVA (Figure 1B, lanes 3 and 5). This is further evidence that processing of HERV-K Gag mediated by the retroviral protease. In addition, MVA-driven protein expression is more efficient than plasmid-based expression, since only one-third of the MVA-infected cell material was loaded in Figure 1B and the infected cells were already harvested 24 hrs after infection.

**Figure 2:**
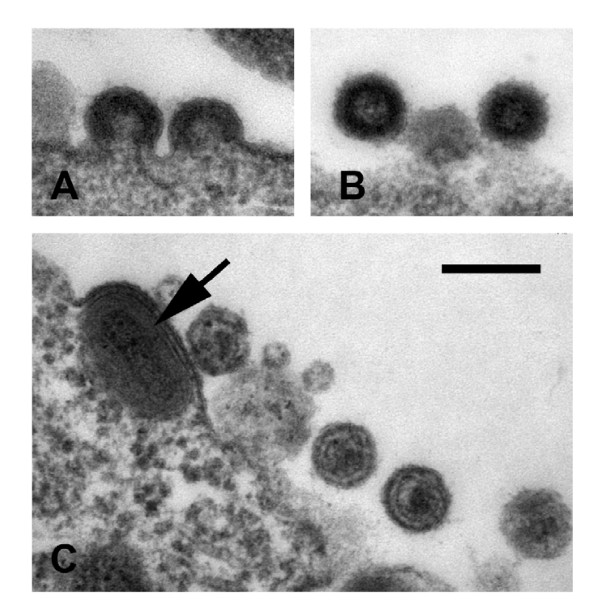
**Morphology of VLPs released from MVA-HERV-K_con _infected cells**. (A) Virus assembly takes place at the cell membrane as is typically seen for C-type viruses. (B) Free immature particles and (C) mature particles with condensed cores could also be observed. Note the MVA particle (arrow) in the HERV-K-producing cell. Bar in (C) represents 200 nm.

To determine if virus-like particles are formed by MVA-HERV-K_con_-infected cells, NIH3T3 cells were infected at an MOI of 5 and the cells were fixed 24 h after infection and analyzed by electron microscopy. Retroviral particles were detected budding at the cell surface (Figure 2A) and Figure 2B shows free immature retroviral particles. Figure 2C shows the presence of incoming poxviral particles as typically seen after MVA infection, as well as mature retroviral particles with condensed cores (Figure 2C). Therefore, supernatants of MVA-HERV-K_con_-infected cells could be used to generate large amounts of HERV-K virus-like particles.

### Separation of processed Gag fragments isolated from VLPs and N-terminal sequence determination

The HERV-K Gag protein has a functional organization typical for retroviruses. *In silico *predictions imply an N-terminal matrix protein (MA) with an N-terminally myristoylated head, which usually directs Gag to cell membranes. At the C-terminus are two Zink-finger motifs, suggesting the location of the nucleocapsid (NC) protein. The NC binds RNA and enables the packaging of the viral genome. The central region of the HERV-K Gag has homology to retroviral capsid (CA) proteins. CA usually homo-oligomerizes and determines the particle morphology. Retroviruses also have other proteins which are generally designated by their molecular weight, such as p12 of murine leukemia virus; however, their function is still unknown [12]. Cleavage of *in vitro*-translated HERV-K Gag by its purified protease expressed in *E. coli *has been shown before [13], but the exact boundaries and the precise size of processed HERV-K Gag proteins were unknown.

**Figure 3:**
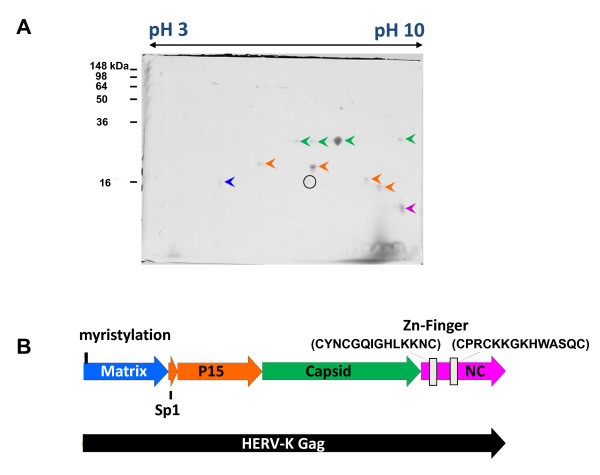
**Mapping of HERV-K Gag protease cleavage sites**. (A) VLPs from MVA-HERV-K_con-_infected cells were pelleted by ultracentrifugation and separated by 2-D gel electrophoresis. The gel was stained with Coomassie and the indicated spots were analyzed by mass spectrometry and N-terminal sequencing. (B) Graphic scheme of HERV-K Gag, the experimentally determined processing products and *in silico *predictions. Colors indicate the analyzed spots isolated by 2-D gel electrophoresis.

To study HERV-K Gag processing, HEK 293T cells were infected at an MOI of 5 for 24 h with MVA-HERV-K_con _and cell supernatants were collected and concentrated by ultracentrifugation through a sucrose cushion. To analyze the Gag cleavage products in VLPs, two-dimensional (2-D) electrophoresis was employed. The concentrated virus-like particles were first subjected to isoelectric focusing, followed by SDS-PAGE as the second dimension (Figure 3). The gel was stained with Coomassie dye and single proteins were cut out and analyzed by mass spectrometry (MS). With the exception of one spot (Figure 3A, indicated by a black circle), all spots analyzed corresponded to HERV-K sequences. As a control, the same procedure was performed with wt MVA-infected cells and, as expected, no proteins were detected in the 2-D analysis (data not shown). Figure 3B summarizes the results, and color coding indicates the regions in HERV-K Gag corresponding to peptide sequences obtained by MS of 2-D separated proteins. Table 1 gives a summary of the peptide fragments obtained by MS analysis. The mass spectrometry did not allow for the identification of the exact cleavage site boundaries. Therefore, we once more purified HERV-K particles from MVA-HERV-K_con_-infected HEK 293T cells and separated the proteins by 2-D electrophoresis and blotted them onto a PVDF membrane. Ponceau S-stained protein spots were cut out and N-terminal sequencing was performed. Table 2 gives a summary of the identified cleavage sites. The isoelectric point (pI) and the molecular weight (MW) were calculated for the protein fragments and correlated with the 2-D analysis (Figure 3A). Consequently, we propose a cleavage pattern of HERV-K as indicated in Figure 3B, with the following fragments:

**Table 1:** Summary of the peptide fragments obtained by MS analysis.

Gag domains	Peptides found by MS analysis (AA position)
Matrix	**_31_**STKNLIKLFQIIEQFCPWFPEQGTLDLK**_58_** **_74_**GNIIPLTVWNDWAIIKAALEPFQTEEDSVSVSDAPGSCIIDCNENTR**_120_**

P15	**_171_**VGPSESKPRGTSPLPAGQVPVTLQPQK**_197_** **_241_**APYPQPPTR**_249_** **_260_**QGSELHEIIDK**_270_**

Capsid	**_324_**QYGPNSPYMRTLLDSIAHGHRLIPYDWEILAKSSLSPSQFLQFKTWWIDGVQEQVR**_379 383_**AANPPVNIDADQLLGIGQNWSTISQQALMQNEAIEQVR**_420_** **_430_**IQDPGSTCPSFNTVRQGSKEPYPDFVAR**_457_** **_499_**VPAGSDVISEYVK**_511_**

NC	**_565_**QNITIQATTTGREPPDLCPR _**584**_ **_602_**NGQPLSGNEQR _**612**_

The amino acid position within the Gag protein and an allocation to the Gag subfragments are indicated.

**Table 2:** Summary of the sequences obtained from N-terminal sequencing of HERV-K Gag cleavage products.

Gag domains	Protease cleavage sites (AA position)		Gag domains
Matrix	LHCEY_134_	_135_VAEPV	SP1-HK
SP1-HK	TQNVD_148_	_149_YNQLQ	P15
P15	EAWQF_282_	_283_PVTLE	Capsid
Capsid	QAITG_532_	_533_VVLGG	Nucleocapsid

- An apparently myristoylated MA of 15.2 kDa, which does not match the calculated pI of 9.1 but is rather shifted to a low pH due to the myristoylation or phosphorylation.

- A short peptide SP1-HK.

- The p15, with a pI of 8.1 and MW of 15.1 kDa.

- The slightly over-presented, uncleaved product of SP1-HK and p15 with a pI of 5.9 and MW of 16.5 running in the middle of the 2-D gel (Figure 3A).

- CA, with a pI of 6.3 and MW 27.5 kDa. The identified N-terminal cleavage site for CA matches the aromatic amino acid/proline sequence at the scissile bond consistently found at the N-terminus of CA proteins of retroviruses.

- NC, with a pI of 9.6 and a MW of 14.6 kDa.

The p15 and the SP1-HK/p15 proteins display two spots in the 2-D analysis. This might result from protein phosphorylation, which changes the pI and can also affect protein mobility in SDS-PAGE analysis. In addition, we detected multiple spots of lower abundance for CA in the 2-D analysis, which might result from post-translational modification, such as phosphorylation [14].

## Discussion

The human genome encodes a large number of endogenous retroviral sequences, which encompass about 8% of the total genome. Human endogenous retroviruses (HERV) are transmitted vertically and are considered to be non-replicating, since almost all HERV genomes have inactivating mutations. Only HERV-K has retained its capability to form viral particles, but there are no signs of infectivity. Little is known about the biological function of HERV-K expression, which has been detected in some tumor entities, such as human teratocarcinomas [4], and the basic virology has still not been investigated properly. In order to elucidate the hypothetical defect in Gag processing and--as a consequence--in virus infectivity, we mapped the previously unknown proteolytic cleavage sites of the HERV-K Gag polyprotein.

HERV-K virus-like particles based on the HERV-K consensus sequence were produced with the help of a recombinant poxvirus expressing the HERV-K Gag, the protease (PR) and the polymerase (Pol), and harvested from supernatants of the infected cells. The production of retrovirus-like particles by vaccinia virus (VACV) expression of the gag/pol genes is well established and has been described previously, for example for HIV-1 [15], and transduction-competent retroviral vectors have also been produced [16]. Moreover, VACV-produced HIV-1 particles have been used to test early HIV protease inhibitors and it could be shown that HIV-1-like particles released from inhibitor-treated cells contained almost exclusively the Gag precursor but no cleaved Gag products [17]. We used the highly attenuated MVA instead of replication-competent VACV which increased the safety of our work and provided proof of concept that MVA is also suitable for producing VLPs for biochemical analysis.

HERV-K particles were released from MVA-HERV-K_con_-infected cells, showing a typical gammaretrovirus morphology with assembly of the virus core at the cytoplasma membrane and extracellular particles of immature and mature morphology, with a condensed, acentric core (Figure 2C), similar to HERV-K113 particles produced by recombinant baculovirus expression [18] or HTDV/HERV-K expressed by teratocarcinoma cell lines [19]. HERV-K Gag proteolytic cleavage was dependent on the presence of its functional protease and potential poxvirus-encoded proteases could not cleave Gag in the absence of the viral protease (Figure 1).

*In silico *prediction of retroviral protease cleavage sites is not possible, because there is no clear consensus sequence motif. The substrate must be in an extended conformation to fit into the active site to be hydrolyzed [20]. Therefore, we identified the cleavage sites by a combination of 2-D electrophoresis, mass spectrometry and N-terminal sequencing, and could draw a map of Gag as illustrated in Figure 3B. The N-terminal cleavage site for HERV-K CA matched the sequence consistently found at the N-terminus of all retroviral CA proteins. The other cleavage sites correspond well to the simplified version of a cleavage site as an amino acid stretch that is hydrophobic and both accessible and flexible, i.e. apparently the space between separately folded domains of Gag. HERV-K Gag is processed into MA, a short spacer peptide, p15, CA and NC. This structure is similar to mouse mammary tumor virus (MMTV). Interestingly, MMTV is morphologically a B-type virus where core assembly takes place inside the cytoplasma and only the preformed core migrates to the cell membrane. In addition, we found late (L)-domain motifs in the p15 protein of HERV-K, similar to Rous sarcoma virus, murine leukemia virus, and Mason-Pfizer monkey virus (MPMV), in which the L-domains also reside between MA and CA. In contrast, the late domains of HIV are located in the C-terminal p6 protein of Gag. L-domains are needed for efficient pinching off of the budding viral particles and different retroviruses utilize different viral proteins to accomplish late budding function [21].

## Conclusion

The retroviral Gag protein is of high importance for retroviruses: it mediates the intracellular transport to the cell membrane, directs assembly of virus particles, and facilitates the budding of the viral particles. Blast alignment of all HERV-K Gag sequences showed that the cleavage sites identified here are highly conserved, and we could not detect a sequence change in the cleavage sites of any of the genomes available in the databases. This suggests that cleavable Gag proteins could be expressed by all HERV-K genomes and that the defect in replication must reside at a different step in the viral life cycle. On the other hand, this also suggests that recombination between HERV-K genomes or other retroviruses like HIV or XMRV [22] might give rise to novel infectious viral genomes able to reinfect human cells.

Note added in proof:

The same cleavage sites were obtained from Norbert Bannert's group (Robert Koch Institute, Berlin) using a different technical approach (George et al., manuscript in preparation).

## Methods

### Cell culture

HEK 293T, BHK21, RK-13 and NIH3T3 cells were cultured in complete Dulbecco's modified Eagle's medium (DMEM) containing 10% fetal bovine serum, penicillin (50 U/ml), streptomycin (50 μg/ml), and L-glutamine (2 mM), and propagated by standard techniques. Transient MVA expressions were performed by transfection of the transfer plasmid and subsequent MVA infection at an MOI of 5. Transfections were performed with Fugene™ (Roche/Mannheim) in 6-well or 10 cm plates according to the manufacturer's instructions. At 24 h post transfection, cells were lysed for SDS-PAGE separation [23], fixed for EM or supernatants were collected for VLP purification. For collection of HERV-K_con _VLPs, HEK 293T cells were infected with MVA-HERV-K_con _at an MOI of 5 for 24 h under serum-free conditions before supernatants were harvested.

### Virus and plasmid construction

The consensus HERV-K gag/pol gene HERV-Gag-PR-Pol [1] was kindly provided by Paul Bieniasz. Sequences were excised by *Not*1 digestion, treated with T4-DNA polymerase to generate blunt ends, and cloned into the blunted *Bam*H1 site of MVA expression plasmid pIII-pmH5 to generate the MVA vector plasmids pIII-HERV-K_con_. The protease-deficient clone pIII-HERV-K_con_pro^- ^was generated via the following cloning steps: HERV-K_con _sequences were excised from pCRVI/Gag-PR-Pol [1] by *Not*1 digestion and cloned into the *Not*1 site of the pCMV-3 Flag plasmid (Agilent Technologies, Waldbronn). Subsequently, the unique *Spe*1 site located upstream of HERV-K PR active center was cut, blunted and religated, resulting in a frameshift and introducing a stop codon. The HERV-K_con_pro^- ^sequence was cut out by *Not*1 and introduced into pIII-mH5 as described above to generate the MVA vector plasmid pIII-HERV-K_con_pro^-^.

The recombinant virus MVA-HERV-K_con _was generated in BHK21 cells by transfection with 1 μg of plasmid DNA, infection at an MOI of 0.05 with MVA-IInew isolate, and plaque selection on RK-13 cells [24], [11]. The recombinant MVA genomes were analyzed by PCR to verify HERV-K gag/pol gene insertion and genetic stability. The production of HERV-K antigens was confirmed by Western blot analysis of various cell lysates harvested after infection with the recombinant MVA-HERV-K_con _(data not shown). Multiple-step growth analysis in chicken embryo fibroblasts (CEFs) demonstrated that the replication capacities of MVA-HERV-K_con _were comparable to non-recombinant MVA (data not shown).

### Ultracentrifugation of cell supernatants

HERV-K VLPs were separated from MVA by filtration through 0.2 μm filters and concentrated by ultracentrifugation of cell supernatants through a 30% sucrose cushion for 2 h at 4°C and 130,000 × g. The viral pellet was resuspended in 150 μl rehydration buffer for 2-D analysis or in 50 μl sample buffer for SDS-PAGE.

### Two-dimensional electrophoresis

HERV-K VLP-containing pellets obtained by ultracentrifugation of MVA-HERV-K_con_-infected HEK 293T cells were resuspended in 150 μl rehydration buffer (8 M urea; 1% CHAPS; 10 mM DTT; 0.25% ampholyte) and used to rehydrate a ReadyStrip IPG Strip pH 3-10 (Bio-Rad, Munich). Samples were isoelectrically focused using a standardized program (20 min 250 V; 2 h 4000 V; 10000 Vh 4000 V). After isoelectric focusing, strips were equilibrated for the second dimension in equilibration buffer (6 M urea; 30% glycerin; 2% SDS; 50 mM Tris pH 8.8) plus 1% DTT (w/v) for 15 min and in equilibration buffer plus 20% iodoacetamide (w/v) for further 15 min. Isoelectrically focused samples were separated via 15% SDS-PAGE with standard techniques followed by Western blot or Coomassie staining for mass spectrometry.

### Western blot

Western blot was performed with a BIO-Rad semi-dry blotter. Proteins separated by SDS-PAGE were blotted onto PVDF membranes with 50 mM sodium borate pH 9.0, 20% methanol, and 0.1% SDS at 100 mA per membrane for 75 min. Afterwards, membranes were either: (1) washed with water and stained with Ponceau S (Sigma Aldrich, Hamburg), and protein spots were cut out and washed extensively with water for N-terminal Edman amino acid sequencing http://proteome-factory.com; or (2) blocked with Roti-Block™ and proteins were detected with α-HERV-K capsid monoclonal antibodies [9] and the ECL detection system (Amersham, Freiburg).

### Electron microscopy

Cells were fixed with 2.5% glutaraldehyde in culture medium for 45 min at room temperature. After washing in PBS, cells were scraped off the culture disk and gently mixed with 2% warm liquid agarose. After cooling and gelling, small agarose blocks containing the cells were cut. These blocks were post-fixed with 2% osmium tetroxide in PBS and treated with 1% tannic acid to improve visibility of viral surface proteins [25]. Cells were then dehydrated in a graded series of ethanol and finally embedded in epoxy resin (Sigma-Aldrich, Steinheim) according to standard preparation protocols. Ultrathin sections were stained with 2% uranylacetate for 15 min followed by 2% lead citrate for 5 min.

### Mass spectrometry

Protein spots of interest were excised from Coomassie-stained 2-D gels, destained, reduced, alkylated and digested with trypsin as described elsewhere [26]. As a modification of this method, peptides were eluted with 25 mM NH_4_HCO_3 _in 10% acetonitrile (ACN) and the digestion was stopped by adding 5% formic acid. The peptides were separated with a nano-Acquity UPLC (Waters, Milford, USA) using a 5 μm symmetry 180 μm × 20 mm c18 pre-column and a 1.7 μm BEH130 100 μm × 100 mm c18 separation column at a flow rate of 500 nl applying a gradient of 30 min (3% ACN to 40% ACN). The UPLC was coupled to a Nano-ESI Synapt mass spectrometer (Waters) operated in V mode, acquiring MS^E ^data and applying standard parameters. Data analysis was performed with the Protein Lynx Global Server Version 2.3 (Waters). Protein hits were accepted at a false positive rate of less than 4%; peptide mass accuracy was 5 ppm or better.

## Competing interests

The authors declare that they have no competing interests.

## Authors' contributions

BK carried out the studies and helped to draft the manuscript; KB performed the EM studies, and AR did the mass spectrometry. BS conceived, designed and coordinated the study and drafted the manuscript. All authors read and approved the final manuscript.

## References

[B1] Lee YN Bieniasz PD Reconstitution of an infectious human endogenous retrovirus PLoS Pathog 2007 3 e10 10.1371/journal.ppat.0030010 17257061 PMC1781480

[B2] Bannert N Kurth R The evolutionary dynamics of human endogenous retroviral families Annu Rev Genomics Hum Genet 2006 7 149 173 10.1146/annurev.genom.7.080505.115700 16722807

[B3] Magin C Lower R Lower J cORF and RcRE, the Rev/Rex and RRE/RxRE homologues of the human endogenous retrovirus family HTDV/HERV-K J Virol 1999 73 9496 9507 10516058 10.1128/jvi.73.11.9496-9507.1999 PMC112984

[B4] Lower R Lower J Frank H Harzmann R Kurth R Human teratocarcinomas cultured in vitro produce unique retrovirus-like viruses J Gen Virol 1984 65 Pt 5 887 898 10.1099/0022-1317-65-5-887 6202829

[B5] Buscher K Trefzer U Hofmann M Sterry W Kurth R Denner J Expression of human endogenous retrovirus K in melanomas and melanoma cell lines Cancer Res 2005 65 4172 4180 10.1158/0008-5472.CAN-04-2983 15899808

[B6] Wang-Johanning F Liu J Rycaj K Huang M Tsai K Rosen DG Chen DT Lu DW Barnhart KF Johanning GL Expression of multiple human endogenous retrovirus surface envelope proteins in ovarian cancer Int J Cancer 2007 120 81 90 10.1002/ijc.22256 17013901

[B7] Herbst H Kuhler-Obbarius C Lauke H Sauter M Mueller-Lantzsch N Harms D Loning T Human endogenous retrovirus (HERV)-K transcripts in gonadoblastomas and gonadoblastoma-derived germ cell tumours Virchows Arch 1999 434 11 15 10.1007/s004280050298 10071229

[B8] Dewannieux M Harper F Richaud A Letzelter C Ribet D Pierron G Heidmann T Identification of an infectious progenitor for the multiple-copy HERV-K human endogenous retroelements Genome Res 2006 16 1548 1556 10.1101/gr.5565706 17077319 PMC1665638

[B9] Bieda K Hoffmann A Boller K Phenotypic heterogeneity of human endogenous retrovirus particles produced by teratocarcinoma cell lines J Gen Virol 2001 82 591 596 11172100 10.1099/0022-1317-82-3-591

[B10] Mueller-Lantzsch N Sauter M Weiskircher A Kramer K Best B Buck M Grasser F Human endogenous retroviral element K10 (HERV-K10) encodes a full-length gag homologous 73-kDa protein and a functional protease AIDS Res Hum Retroviruses 1993 9 343 350 10.1089/aid.1993.9.343 8512750

[B11] Staib C Drexler I Sutter G Construction and isolation of recombinant MVA Methods Mol Biol 2004 269 77 100 15114009 10.1385/1-59259-789-0:077

[B12] Lee SK Nagashima K Hu WS Cooperative effect of gag proteins p12 and capsid during early events of murine leukemia virus replication J Virol 2005 79 4159 4169 10.1128/JVI.79.7.4159-4169.2005 15767417 PMC1061564

[B13] Schommer S Sauter M Krausslich HG Best B Mueller-Lantzsch N Characterization of the human endogenous retrovirus K proteinase J Gen Virol 1996 77 Pt 2 375 379 10.1099/0022-1317-77-2-375 8627242

[B14] Gottwein E Krausslich HG Analysis of human immunodeficiency virus type 1 Gag ubiquitination J Virol 2005 79 9134 9144 10.1128/JVI.79.14.9134-9144.2005 15994808 PMC1168789

[B15] Karacostas V Nagashima K Gonda MA Moss B Human immunodeficiency virus-like particles produced by a vaccinia virus expression vector Proc Natl Acad Sci USA 1989 86 8964 8967 10.1073/pnas.86.22.8964 2479031 PMC298411

[B16] Konetschny C Holzer GW Urban C Hammerle T Mayrhofer J Falkner FG Generation of transduction-competent retroviral vectors by infection with a single hybrid vaccinia virus J Virol 2003 77 7017 7025 10.1128/JVI.77.12.7017-7025.2003 12768020 PMC156191

[B17] McQuade TJ Tomasselli AG Liu L Karacostas V Moss B Sawyer TK Heinrikson RL Tarpley WG A synthetic HIV-1 protease inhibitor with antiviral activity arrests HIV-like particle maturation Science 1990 247 454 456 10.1126/science.2405486 2405486

[B18] Tonjes RR Boller K Limbach C Lugert R Kurth R Characterization of human endogenous retrovirus type K virus-like particles generated from recombinant baculoviruses Virology 1997 233 280 291 10.1006/viro.1997.8614 9217052

[B19] Lower R Lower J Kurth R The viruses in all of us: characteristics and biological significance of human endogenous retrovirus sequences Proc Natl Acad Sci USA 1996 93 5177 5184 10.1073/pnas.93.11.5177 8643549 PMC39218

[B20] Vogt VM Proteolytic processing and particle maturation Curr Top Microbiol Immunol 1996 14 95 131 10.1007/978-3-642-80145-7_4 8791726

[B21] Martin-Serrano J The role of ubiquitin in retroviral egress Traffic 2007 8 1297 1303 10.1111/j.1600-0854.2007.00609.x 17645437

[B22] Enserink M Chronic fatigue syndrome. New XMRV paper looks good, skeptics admit--yet doubts linger Science 2010 329 1000 10.1126/science.329.5995.1000 20798285

[B23] Schnierle BS Moritz D Jeschke M Groner B Expression of chimeric envelope proteins in helper cell lines and integration into Moloney murine leukemia virus particles Gene Ther 1996 3 334 42 8732165

[B24] Staib C Lowel M Erfle V Sutter G Improved host range selection for recombinant modified vaccinia virus Ankara Biotechniques 2003 34 694 6 12703290 10.2144/03344bm02

[B25] Gelderblom HR Hausmann EH Ozel M Pauli G Koch MA Fine structure of human immunodeficiency virus (HIV) and immunolocalization of structural proteins Virology 1987 156 171 176 10.1016/0042-6822(87)90449-1 3643678

[B26] Albrecht M Alessandri S Conti A Reuter A Lauer I Vieths S Reese G High level expression, purification and physico- and immunochemical characterisation of recombinant Pen a 1: a major allergen of shrimp Mol Nutr Food Res 2008 52 Suppl 2 S186 S195 18727010 10.1002/mnfr.200700424

